# Human Menopausal Gonadotropin versus Recombinant FSH in Polycystic Ovary Syndrome Patients Undergoing *In Vitro* Fertilization

**Published:** 2013-03-03

**Authors:** Ayse Figen Turkcapar, Berna Seckin, Gogsen Onalan, Tulin Ozdener, Sertac Batioglu

**Affiliations:** 1 Department of Reproductive Endocrinology, Zekai Tahir Burak Women’s Health Education and Research Hospital, Ankara, Turkey; 2 Department of Obstetrics and Gynecology, Baskent University School of Medicine, Ankara, Turkey

**Keywords:** hMG, Recombinant FSH, *in vitro* Fertilization, Polycystic Ovary Syndrome

## Abstract

**Background:**

We aimed to compare human menopausal gonadotropin (hMG) and recombinant follicle-stimulating hormone (r FSH) with respect to clinical outcomes and the development of ovarian hyperstimulation syndrome (OHSS) for patients with polycystic ovary syndrome (PCOS) treated with *in vitro* fertilization (IVF).

**Materials and Methods:**

This prospective randomized controlled trial included a total of 80 women with PCOS. Of these, 38 were randomized to receive treatment with hMG and 42 with rFSH using a long gonadotropin releasing hormone (GnRH) analogue protocol. Outcome measures were cycle characteristics, pregnancy rates, the need for coasting, and OHSS rates.

**Results:**

In the hMG group we observed a significantly lower peak estradiol (E2) level (p=0.02), fewer intermediate-sized follicles (p=0.001), lower number of oocytes retrieved (p=0.002) and metaphase II (MII) oocytes (p=0.003). However, there were no significant differences between the groups in the number of fertilized oocytes, fertilization rates, top quality embryo counts, and the number of transferred embryos. There was no difference in pregnancy rates between the groups. OHSS occurred in 11.9% of the rFSH group patients, whereas no OHSS developed in the hMG group. Coasting requirements were lower in the hMG group (19.2% vs. 48.9%, p=0.013).

**Conclusion:**

Ovarian stimulation with hMG and rFSH provides similar clinical pregnancy rates in PCOS patients treated with a long GnRH agonist protocol in IVF cycles. hMG stimulation appears to be associated with a lower rate of OHSS and decreased coasting requirements (Registration Number: NCT01365936).

## Introduction

Obtaining multi-follicular growth is the goal of ovarian stimulation for assisted reproductive technologies (ART). One of the most severe complications of ovarian stimulation is ovarian hyper stimulation syndrome (OHSS), which occurs in 1-10% of *in vitro* fertilization (IVF). cycles. Polycystic ovary syndrome (PCOS) patients are at high risk of developing OHSS (1). In this particular group of patients it is important to use an ovarian stimulation agent which is safe and effective enough to obtain optimum clinical outcomes during IVF cycles.

PCOS is the most common disorder that causes chronic anovulation in the infertile population with persistently elevated estrogen and luteinising hormone (LH) levels (2). The role of LH in folliculogenesis is more complex and somewhat divergent. During folliculogenesis, while follicle stimulating hormone (FSH) stimulates the recruitment and growth of the preantral-small antral follicles, LH supports the selection and growth of dominant follicles and atresia of cohorts of small follicles stimulated by FSH (3). This physiologic atretic effect of LH may exploit the monofollicular growth in PCOS patients. Preliminary data in PCOS patients that have been treated with recombinant FSH (rFSH) for non-ART ovulation induction suggest that rLH can hasten small ovarian follicle demise and allow for selective achievement of monofolliculogenesis (3). This could lead to a reduction in the risk of ovarian hyperstimulation.

The use of gonadotropin during controlled ovarian hyperstimulation of PCOS patients is a major challenge. There have been some controversies regarding the use of preparations with LH activity in PCOS women. The use of FSH-only products rather than human menopausal gonadotropin (hMG) in PCOS, where endogenous LH is already elevated, is expected to have theoretical advantage and has been advocated in this group of women (4). To the best of our knowledge, there are no studies in the literature which compare the use of hMG and rFSH for patients with PCOS in IVF treatment.

Therefore, the aim of this study was to compare urinary hMG with rFSH in PCOS patients for clinical outcomes and OHSS rates in IVF treatment cycles.

## Materials and Methods

This prospective randomized controlled trial was conducted between January 2008-December 2008 in Zekai Tahir Burak Women’s Health Education and Research Hospital. The study was approved by the hospital Ethics Committee. The protocol was explained to the patients before they entered the study and informed consent was obtained from each couple. PCOS was diagnosed according to the revised Rotterdam criteria by the European Society for Human Reproduction/American Society of Reproductive Medicine (ASRM) as the presence of oligo-and/or anovulation and sonographically confirmed polycstic ovaries (5). Exclusion criteria were as follows: females older than 39 years or serum FSH levels >12mIU/mL, history of ovarian surgery and/or the presence of severe male infertility that required testicular sperm extraction. All patients were treated with oral contraceptive pills (Yasmin, Scherring, Germany) during the cycle preceding ovulation induction. Leuprolide acetate (Lucrin Daily, Abbott Cedex, Istanbul, Turkey) therapy was started in the mid-luteal phase at an initiation dose of 1.0 mg subcutaneous (SC) daily until pituitary down-regulation was established. After gonadotropin releasing hormone (GnRH) analogue suppression was achieved with an endometrial thickness <5 mm and serum estradiol (E2) level <45 pg/mL, the leuprolide acetate dose was reduced to 0.5 mg daily. For ovarian stimulation, we randomized patients to one of the following treatments: hMG (Menogon, Ferring Pharmaceuticals, Istanbul, Turkey) or rFSH (Gonal-F, Serono, Istanbul, Turkey) with an initial 150 IU daily dose. Gonadotropin stimulation treatment assigned to each patient was determined according to a computer-generated randomization list. Gonadotropin dosage was adjusted accordingly by serum E2 levels and sonographic findings. Human chorionic gonadotropin (hCG, Pregnyl, Organon, the Netherlands) at a dose of 5.000 IU intramuscular (IM) was administrated when at least three follicles reached a mean diameter of 18 mm. The criteria for coasting in our institute were the presence of at least 20 follicles, each measuring ≥10 mm in diameter , of which ≥20% of these follicles had diameters ≥15 mm and serum E2 levels >3600 pg/mL. During the coasting period, gonadotropin was withheld and leuprolide acetate was continued at 0.5 mg/d. Blood samples were taken daily until serum E_2_ levels decreased to ≤4000 pg/mL when hCG was administered. Transvaginal oocyte retrieval was scheduled 36 hours after the hCG injection. Intracytoplasmic sperm injection was performed for all metaphase II (MII) oocytes per our clinical policy. Fertilization was assessed at 20 ± 1 hour and embryo quality was assessed at 28, 44 and 68 hours (±1 hour) after oocyte retrieval. We defined a top-quality embryo as one that had four cells on day 2 and eight cells on day 3, with no multinucleation and fragmentation. A maximum of four embryos were transferred at two or three days after oocyte retrieval. This study was conducted before the new legislation that limited the number of embryos to be transferred in our country. Therefore, multiple embryos were transferred during this study period.

For luteal support, vaginal progesterone gel (Crinone 8%, Merk Serono, Germany) at a dose of 90 mg/day was given from the time of oocyte retrieval until clinical pregnancy (9-10 weeks of gestation) or negative serum β-hCG test (13-15 days after embryo transfer). Clinical pregnancy was defined as the presence of a gestational sac with accompanying fetal heart beat as observed by ultrasound. OHSS was diagnosed and classified as described by the Practice Committee of the ASRM (6).

For patients, we determined the cycle characteristics of serum peak E2 levels; endometrial thickness on the day of hCG injection; duration of stimulation; total dose of gonadotropins used; number of follicles ≥14 mm and 10-14 mm; number of retrieved, MII and fertilized oocytes; number of top quality and transferred embryos; as well as clinical pregnancy and take home baby rates per cycle, the need for coasting, and the incidence of OHSS.

Statistical analysis of the data was performed using SPSS for Windows v. 11.5 statistical package program (SPSS, Chicago, IL). The Shapiro- Wilk test was used to test for normal distribution of continuous data. If the normality assumption for the comparison of means between two groups was satisfied, we used the student’s t-test for the comparisons of means. Alternatively, if there was evidence of non-normality, the Mann- Whitney test was used. Comparisons between proportions were performed with Pearson’s chisquare or Fisher’s exact tests. All reported p-values were two-tailed and statistical significance was set at 0.05.

## Results

We included 84 women with PCOS treated in the IVF unit of a tertiary referral hospital in this study, of which four women were lost to follow-up. Thus, 80 women completed the study, 38 patients in the hMG group and 42 patients in the rFSH group.

Patients’ characteristics revealed no significant differences between the groups for age, body mass index and baseline hormone levels, which confirmed the appropriate randomization (Table 1).

**Table 1:** Patient characteristics in the treatment groups


Variable	hMG (n=38)	rFSH (n=42)	P value

**Age (Years)**	25.85 ± 3.92	25.98 ± 3.92	0.883
**Body mass index(kg/m^2^)**	25.85 ± 4.90	25.08 ± 4.38	0.527
**Basal FSH(mIU/mL)**	5.63 ± 2.41	6.31 ± 1.58	0.087
**Basal LH(mIU/mL)**	5.86 ± 2.41	6.41 ± 4.54	0.556


Values are given as mean ± SD and p< 0.05 is considered significant.

**Table 2:** Cycle characteristics and outcomes of patients in the treatment groups


	hMG (n=38)	rFSH (n=42)	P value

**Duration of gonadotropin stimulation (Days)**	11.46 ± 1.90a	10.36 ± 1.58	0.025*
**Total dose of gonadotropin (IU)**	1716.06 ± 511.52	1429.50 ± 340.54	0.57
**Peak E2 (pg/mL)**	2880.23 ± 1284.22	3779.52 ± 1487.70	0.02*
**Number of mature follicles (≥14 mm)**	5.92 ± 2.72	6.24 ± 3.85	0.75
**Number of intermediate sized follicles (10-14 mm)**	9.35 ± 3.61	12.69 ± 3.98	0.001*
**Endometrial thickness (mm)**	10.54 ± 2.03	11.45 ± 1.85	0.06
**Number of oocytes retrieved**	9.54 ± 4.31	13.60 ± 5.56	0.002*
**Number of MII oocytes**	7.65 ± 3.39	11.20 ± 5.06	0.003*
**Percentage of MII oocytes**	81.24 (40-100)b	82.13 (37.5-100)	0.80
**Number of oocytes fertilized**	4.46 ± 2.62	6.07 ± 3.55	0.70
**Fertilization rate (%)**	56.95	55.53	0.77
**Number of top quality embryos**	1.29 (0- 3)	1.48 (0-3)	0.48
**Number of embryos transferred**	3 (1-4)	3 (1-4)	0.25
**Clinical pregnancy rate per cycle (%)**	23.1	40.5	0.14
**Coasting requirement (%)**	19.2	48.9	0.013*
**OHSS (n,%)**	0 (0.0%)	5 (11.9%)c	0.14
**Take home baby rate per cycle (%)**	23.1	35.7	0.27


a; Mean ± SD , b; Median (range), c; Number (percentage) and *; p< 0.05 is considered significant.

The mean duration of gonadotropin stimulation was significantly longer in the hMG group (11.46 ± 1.90 vs. 10.36 ± 1.58 days, p=0.025). There was no significant difference in total dose of gonadotropins between the groups. The mean mature follicle (≥14 mm) count was similar, but the mean intermediatesized follicle (10-14 mm) count was significantly lower in the hMG group (9.35 ± 3.61) compared to the rFSH group (12.69 ± 3.98, p=0.001). The mean peak E2 level was significantly lower in hMG group (2880.23 ± 1284.22 pg/mL) compared to the rFSH group (3779.52 ± 1487.70 pg/mL, p=0.02). Also, the mean number of oocytes retrieved were significantly lower in the hMG group (9.54 ± 4.31) compared to the rFSH group (13.60 ± 5.56, p=0.002). MII oocytes were significantly lower in the hMG group (7.65 ± 3.39) compared to the rFSH group (11.20 ± 5.06, p=0.003). There were no significant differences between groups with regards to endometrial thickness, percentage of MII oocytes, number of fertilized oocytes, fertilization rates, top quality embryo counts, and the number of transferred embryos.

Coasting requirement was significantly lower in the hMG group (19.2% vs. 48.9%, p=0.013). The need for coasting longer than three days was not required for any patient. OHSS rate was 11.9% (5 patients) in the rFSH group, whereas no patient developed OHSS in the hMG group, but this was not significant (p=0.14). All hyperstimulation cases were mild. Moderate or severe OHSS was not observed in either group. The clinical pregnancy and take home baby rates were similar in both groups (Table 2).

## Discussion

The occurrence of small-sized preovulatory ovarian follicles is directly related to FSH stimulation and leads to ovulation induction complications. The small/medium-sized follicles are mostly responsible for high serum E2 concentrations and vasoactive compounds leading to OHSS (7). The possibility of selectively inducing atresia in this follicle population without altering the delicate equilibrium with larger, mature follicles is the key step in coasting and prevention of OHSS. As a consequence, this results in reductions in serum E2 levels, vascular endothelial growth factor (VEGF), and other vasoactive mediators; however oocyte yield and cycle outcome will not be affected. The demise of a cohort of smaller follicles with the use of LH during folliculogenesis creates avenues for new therapeutic possibilities. The atretic effect of LH in small/medium follicles will induce developmental arrest while driving the final stages of folliculogenesis in pre-ovulatory follicles, as has been shown recently (3, 8).

Platteau et al. (9) have reported that stimulation with highly purified-hMG (HP-hMG) in anovulatory women in non-IVF cycles is associated with ovulation rates at least as good as rFSH and a lower incidence of OHSS. They suggest that LH activity modifies follicular development and decreases the number of intermediate sized follicles, which could result in a safer, more controlled stimulation cycle. Similar results have been reported in a study by Smitz et al. (10). According to their findings, the presence of LH activity in the HPhMG preparation results in a more selective follicle recruitment process than the FSH-only gonadotropin. Loumaye et al. (11) have also assessed the impact of LH on follicular growth during the late follicular phase in anovulatory patients. They stated that rLH alone can trigger follicular growth arrest, which suggested the existence of an “LH ceiling” during late follicular maturation and have hypothesized that there might be a potential benefit of the usage of LH in ovarian stimulation regimens to promote mono-ovulation. Another report on the consequences of LH activity on folliculogenesis has been reported by Hugues et al. (12). In their study, in patients who over-responded to FSH during ovulation induction, administration of rLH in the late follicular phase appeared to increase the proportion of patients who developed a single dominant follicle. Thus, according to these researchers, the use of LH-containing preparations such as hMG in ovulation induction might be advantageous in the protection from OHSS. Our findings have confirmed these earlier studies. We found that patients who used hMG had significantly lower serum estradiol levels and fewer intermediate sized follicles, which might explain the reduced incidence of OHSS and coasting requirements compared to the rFSH group in PCOS patients. We observed no OHSS in the hMG group, but the difference between the groups did not reach the level of significance (p=0.14), which was probably due to the small number of OHSS cases. Furthermore, ovarian stimulation with hMG and rFSH provided similar clinical pregnancy rates.

Although the number of oocytes retrieved and the number of MII oocytes were lower in the hMG group, the percentage of MII oocytes, fertilization rate, number of top quality embryos and number of embryos transferred were comparable between the two groups in our study. It has been considered that quality is more important than quantity; the success criterion has changed from obtaining many oocytes to obtaining an adequate cohort of top-quality embryos. In a study by Andersen et al. (13), more oocytes were obtained with rFSH than with HP-hMG, but they stated that this increased number of oocytes was not accompanied by a higher number of top-quality embryos. Actually, the proportion of top-quality embryos was significantly higher in the HP-hMG group.

In recent years, rFSH has increasingly been used in ovulation induction and IVF treatments. A number of studies have evaluated the effectiveness of rFSH and hMG in IVF cycles (14-16). A Cochrane review reported the clinically relevant outcomes of ongoing pregnancies or live births (17). Recently, a meta-analysis of compromising true randomized controlled trials showed an equivalent clinical efficacy of these two preparations (18). In the results of the current systematic review, hMG has been demonstrated to be superior to r FSH with regard to clinical outcomes, without increasing the changes of ovarian hyperstimulation (19). Likewise, in a systematic review of randomized trials, Coomarasamy et al. (16) showed a significant increase of 4% in live birth rate with the use of hMG compared with rFSH following a long down-regulation protocol in IVF- intracytoplasmic sperm injection (ICSI) treatment cycles. However, in all of these studies PCOS patients were excluded and the only main outcome measures were live birth and ongoing pregnancy rates. In a recent study by Torabizadeh (20), the outcomes of IVF treatment in PCOS patients with different ovulation methods such as FSH, hMG or their combination were compared. With regard to fertility outcome there were no differences observed, however this study did not investigate the OHSS rate.

To the best of our knowledge, this is the first prospective design study evaluating PCOS patients in IVF treatment with the outcome measures of pregnancy and OHSS rates. The main limitation of our study is its size. Ovulation induction with rFSH is common in PCOS women. Since women with PCOS already have elevated endogen LH levels, the use of hMG is not preferred in our clinics as with other IVF centers. For this reason, this study has been conducted with a restricted patient population. As no data on this issue are currently available, this study may be considered a feasibility study.

## Conclusion

Based on the current study, ovarian stimulation performed with hMG in PCOS patients treated with a long GnRH agonist protocol results in the same clinical pregnancy and take baby home rates compared to ovarian stimulation with rFSH. However, for the consideration of other important factors such as the need of coasting and safety in particular, hMG has major advantages over rFSH. This results warrant further evaluation in a larger prospective series.
